# In vitro and in vivo investigation of the antibacterial, antivirulence, and antiquorum sensing activities of β-carotene against difficult-to-treat resistant *Pseudomonas aeruginosa*

**DOI:** 10.1186/s12866-025-04234-7

**Published:** 2025-08-12

**Authors:** Khaled B. Al-Monofy, Ahmed A. Abdelaziz, Amal M. Abo-Kamar, Lamiaa A. Al-Madboly, Mahmoud H. Farghali

**Affiliations:** Department of Microbiology and Immunology, Faculty of Pharmacy, Tanta University, Tanta, Egypt

**Keywords:** DTR *P. aeruginosa*, Antipseudomonal, β-Carotene, Antivirulence, Antibiofilm, Quorum sensing

## Abstract

**Background:**

Difficult-to-treat resistant (DTR) *Pseudomonas aeruginosa* has strong drug resistance and can tolerate a diversity of antibiotics. Infections triggered by DTR *P. aeruginosa* urgently require research and development of innovative antibiotics. β-Carotene is a carotenoid pigment with multiple activities, such as antibacterial, antifungal, antioxidant, and anticancer activities, therefore, the current study aimed to investigate the potential antipseudomonal activity of β-carotene against DTR *P. aeruginosa* to find a new treatment option.

**Methods:**

Antimicrobial susceptibility tests were conducted for 100 *P. aeruginosa* isolates. Well diffusion and broth microdilution techniques were utilized to determine the antibacterial activity of β-carotene. A total of 10 virulence factors (biofilm, pyocyanin, motility, protease, gelatinase, exopolysaccharide, siderophore, hemolysin, pyomelanin, and rhamnolipid) were screened among the tested isolates, and the effect of β-carotene on these virulence factors was assessed. Light microscopy and confocal laser scanning microscopy approaches were established to investigate the effect of β-carotene on biofilm formation by DTR isolates. Molecular analysis, docking study, and the wound infection model further evaluated the antipseudomonal action of β-carotene against DTR isolates.

**Results:**

All tested multidrug-resistant (MDR) and DTR isolates (*n* = 40) were susceptible to β-carotene (200 µg/ml), with inhibition zones ranging from 10 to 33 mm, recording minimum inhibitory concentration (MIC) values of 12.5 to 100 µg/ml. It was reported that β-carotene reduced the production of all tested virulence factors by DTR isolates, and the percentages of inhibition ranged from 21.5 to 100%. Additionally, β-carotene-treated biofilms showed a significant reduction in biomass and thickness (up to 60%). Moreover, the production of virulence-associated genes, namely *lasR*, *rhlR*, and *pqsR* genes, was downregulated by 0.5 MIC of β-carotene. The molecular docking displayed a resilient LasR-β-carotene complex with a binding free energy of -8.6 kcal/mol. The β-carotene-treated rat group showed accelerated wound closure (up to 90% at day 6) and lower *Pseudomonas* burden (3.3 colony forming unit/g). In addition, the histological examination showed re-epithelialization in the epidermis layer with a few capillaries and newly formed hair follicles in the treated group.

**Conclusion:**

This is the first study to report the antibacterial, antivirulence, and antiquorum sensing activities of β-carotene against DTR *P. aeruginosa*, signifying the promising role of β-carotene in mitigating DTR *P. aeruginosa*-caused infections.

## Introduction

Many acute and chronic infections in humans are caused by the Gram-negative bacterium *Pseudomonas aeruginosa* [1]. More than 500,000 deaths were attributed to *P. aeruginosa* in 2019, making it a major source of nosocomial infections worldwide [2]. *P. aeruginosa* that is not susceptible to at least one antimicrobial agent in three or more antimicrobial classes that are active against this pathogen is classified as multidrug-resistant (MDR) [3]. In the case of difficult-to-treat resistant (DTR) *P. aeruginosa*, it is non-susceptible to levofloxacin, ciprofloxacin, piperacillin-tazobactam, meropenem, aztreonam, ceftazidime, imipenem–cilastatin, and cefepime [4]. The infections caused by DTR *P. aeruginosa* in healthcare settings were dramatically increased [5], and controlling these infections represents a substantial challenge [3].

According to Kunwar et al. (2021), *P. aeruginosa* is a typical biofilm-forming opportunistic bacterium [6]. Biofilm is a structure primarily composed of autogenic extracellular polymeric materials that serve as a scaffold to hold bacteria together on surfaces, shield them from environmental stressors, and prevent phagocytosis, allowing for colonization and long-term persistence [7]. Additionally, *P. aeruginosa* possesses a number of virulence agents, such as siderophore, pyocyanin, protease, and rhamnolipids [8]. These virulence factors are essential for the host’s pathogenesis and can directly or indirectly lead to resistance to various antibacterial treatments [9].

*P. aeruginosa* uses quorum sensing (QS), a cell-to-cell communication system, to regulate its behavior and virulence by releasing particular signaling molecules called autoinducers (AI) [10]. To accomplish this communication, *P. aeruginosa* notably uses four different QS systems. The LasR/I system is the primary QS and generates the 3O-C12-HSL as AI [10]. This AI binds to the LasR protein, which activates the expression of LasI, induces the production of virulence factors, and triggers the formation of biofilms [11]. Knowing this complex network of QS in *P. aeruginosa* enables us to better understand how this bacterium develops antibiotic resistance and causes infection. Anti-virulence approaches centered on QS and the prevention of biofilm formation are the primary fields of attention for researchers looking into novel ways to fight *P. aeruginosa* infections [12].

β-Carotene is prominent member of the carotenoid family with a molecular weight of 536.88 and chemical formula C_40_H_56_ [13]. The antimicrobial properties of β-carotene have been documented in numerous studies against various microbial species, such as *Staphylococcus aureus*, *Streptococcus agalactiae*, *Klebsiella pneumoniae*, *Fusarium oxysporum*, *Aspergillus niger*, and *Penicillium chrysogenum* [14–16]. β-Carotene’s antimicrobial effect is linked to its capacity to dissolve in the lipids of the plasma membrane, increasing penetration through the membrane that leads to inhibiting or destroying the growth of some pathogenic microorganisms [16]. Therefore, the goal of the current study was to examine the potential antipseudomonal action of β-carotene against DTR *P. aeruginosa* by examining its effect on bacterial growth, virulence factors, and QS.

## Materials and methods

### Sample collection

The tested isolates (150 isolates) were obtained from the microbiological laboratory at Tanta University Hospital in Egypt. Then, the isolates were identified by culturing on *Pseudomonas* selective medium, Cetrimide agar, and by a panel of biochemical examinations [17].

### Antimicrobial susceptibility testing (AST)

The AST was carried out for the identified *P. aeruginosa* isolates (*n* = 100) on Mueller-Hinton agar plates using the Kirby-Bauer disk diffusion method, according to European Committee on Antimicrobial Susceptibility Testing (EUCAST) 2022 standards and Clinical and Laboratory Standards Institute (CLSI) 2021. The tested antibiotics were ceftazidime (10 µg), cefepime (30 µg), meropenem (10 µg), ciprofloxacin (5 µg), levofloxacin (5 µg), gentamicin (10 µg), aztreonam (30 µg), polymyxin B (300unit), tobramycin (10 µg), and piperacillin/tazobactam (100 + 10 µg). The AST for imipenem/cilastatin and colistin was performed using the broth microdilution method [18]. The MDR (not susceptible to at least one agent in ≥ 3 antibiotic groups) and DTR (not susceptible to levofloxacin, ciprofloxacin, piperacillin-tazobactam, meropenem, aztreonam, ceftazidime, imipenem–cilastatin, and cefepime) isolates were selected for our study [4, 19]. These isolates were selected as we specifically aimed to examine the potential antipseudomonal activity of β-carotene against resistant *P. aeruginosa* isolates to find a new treatment option, as the current treatment choices become limited. The selected isolates’ multiple antibiotic resistance (MAR) index was calculated by dividing the total number of detected resistances to antimicrobials for each isolate by the total number of tested antimicrobials [20]. *Pseudomonas aeruginosa* ATCC 27,829 used as a reference strain.

### Antibacterial activity and minimum inhibitory concentration (MIC)

The antimicrobial activity of β-carotene against MDR/DTR isolates was performed via a well-diffusion method. A volume of 100 µl of 0.5 McFarland tested isolate was spread on Mueller-Hinton agar plates, and β-carotene (100 µl), purchased from Sigma-Aldrich and prepared by dissolving the powder in 10% dimethyl sulfoxide (DMSO), was added to the wells (6 mm diameter) at a concentration of 200 µg/ml. Following the 24 h incubation at 37 °C, the diameter of the inhibition zone for each isolate was measured. A negative control was 10% DMSO [21]. The MIC of β-carotene against MDR/DTR isolates was performed via the broth microdilution method in Mueller-Hinton broth at concentrations ranging from 1.5625 to 100 µg/ml. The values of MIC were determined after incubation at 37 °C for 24 h by visually investigating the microplate against a black background; the lowest concentration with undetectable growth was considered MIC [22]. *P. aeruginosa* ATCC 27,829 used as a reference strain.

### Growth curve analysis

The DTR isolates were inoculated into double-strength Luria-Bertani (LB) broth (optical density (OD) at 630 nm = 0.3), and a volume of 100 µl was deposited in the wells of a microtiter plate. After that, β-carotene solution (100 µl) was added at 0, 0.5, and 1 MICs and mixed by aspiration using a micropipette. The ODs were measured at 0 h, 2 h, 4 h, 6 h, and 12 h via measuring OD at 630 nm using a microplate reader (Sunrise™, TECAN, Switzerland). The measured ODs were plotted against time (h) to elucidate the effect of β-carotene at 0.5 and 1 MICs on the growth of DTR *P. aeruginosa* isolates [23].

## Screening of virulence factors of MDR/DTR *P. aeruginosa* and estimation of antivirulence activity of β-carotene against DTR *P. aeruginosa*

### Biofilm formation

The isolates were incubated in LB broth supplemented with 1% glucose (10^6^ colony forming unit (CFU)/ml) using a 96-well plate for 24 h at 37 °C. The broth and planktonic cells were removed, and then the wells were washed with phosphate-buffered saline (PBS). After the plate drying, the remaining attached biofilms were stained with 0.1% crystal violet (200 µl), and after 15 min, the leftover stain was removed via washing with sterile water. About 200 µl of glacial acetic acid (33% vol/vol) was added to liquefy the biofilms that were evaluated by a microtiter reader (Sunrise™, TECAN, Switzerland) by assessing the absorbance at 595 nm [24]. The effect of β-carotene on the formation of biofilms by DTR isolates was estimated using the method conducted by Kim et al. (2022) [24]. After the preparation of bacterial suspension as mentioned above, a volume of 20 µl of bacterial suspension and 180 µl of medium containing β-carotene (0.5 MIC) were added to the microplate, except for the control, where the medium was added without β-carotene, and then incubated for 24 h at 37 °C. The formation of biofilms with and without β-carotene was measured via crystal violet stain as described above, and the percentage of inhibition was evaluated according to the following formula: [(OD(control) − OD(test)/OD (control)] ×100.

### Pyocyanin production

The tested isolates were incubated in LB broth (0.5 McFarland) for 24 h at 37 °C. After that, the broth was centrifuged at 10,000 rpm for 10 min to remove the bacterial cells, and the pyocyanin was quantified in the supernatant by measuring the absorbance at 691 nm (λmax of pyocyanin) using a UV-Vis spectrophotometer (Genesys™ 10 S, Thermo Scientific, WI, USA). In the case of examining the inhibitory effect of β-carotene on pyocyanin, DTR isolates were incubated in LB broth with 10% DMSO, a negative control, and in LB broth with β-carotene (0.5 MIC) for 24 h at 37 °C. After 10 min of centrifugation at 10,000 rpm, the absorbance of supernatants of treated and untreated bacteria was assessed at 691 nm [25].

### Swimming motility measurement

The motility of tested isolates was determined using the tryptone swimming plates as described by Kamer et al. (2023) [26]. The isolates were stab incubated at 37 °C. After 24 h of incubation, the migration of colonies from the inoculation point was measured, representing the swimming diameter. The inhibitory effect of β-carotene on motility was evaluated by incubation of DTR isolates in LB broth and β-carotene (0.5 MIC)-containing LB broth for 24 h at 37 °C. Then, the treated and untreated cells were spot inoculated on tryptone swimming plates, and the motility was measured after incubation at 37 °C for 24 h [26].

### Protease production

Using skim milk agar plates (1.5% LB agar containing 5% skimmed milk), the isolates were incubated at 28 °C for 48 h. Then, the proteolytic activity was evaluated by the presence of a clear lysis zone around the bacterial growth [27]. In case of screening the effect of β-carotene on proteolytic activity, the DTR isolates were incubated in LB broth with and without β-carotene (0.5 MIC) for 24 h at 37 °C, a volume of 100 µl of filtered (0.45 μm filter) supernatant of treated and untreated bacteria was added to the wells of skim milk agar plates. The formed zones were assessed after 24 h of incubation at 37 °C [28]. Additionally, a quantitative protease assay was conducted using Folin-Ciocalteu reagent. In brief, a volume of 2 ml of cell-free supernatant from both treated and untreated bacteria was mixed with 2 ml of casein solution (0.65% casein w/v in a 50 mmol potassium phosphate buffer, pH 7.5). The mixture was then incubated for 10 min at 37 °C, and then 5 ml of 110 mmol trichloroacetic acid was added to terminate the reaction at room temperature for 30 min. Following centrifugation at 5000 rpm for 15 min, 2 ml of the filtrate was mixed with 5 ml of 500 mmol sodium carbonate and 2 ml of Folin-Ciocalteu reagent. The absorbance was measured at 660 nm, and the percentage inhibition in protease production was calculated [29].

### Gelatinase production

By means of gelatine agar plates, the tested isolates were streaked and incubated for 24 h at 28 °C. The gelatinase-producing isolates were identified by forming a clear zone around the bacterial growth after the addition of Frazier’s solution (15 g of HgCl_2_ solubilized in 20 ml of 37% HCl, then distilled water was added to 100 ml) to precipitate the intact gelatin [30]. The gelatinase inhibitory effect of β-carotene was tested by 24 h incubation at 37 °C of the tested isolates in LB broth with and without β-carotene (0.5 MIC). After centrifugation, the obtained supernatants (100 µl) were added to the formed wells in gelatine agar plates. The gelatine hydrolysis zones after 24 h of incubation at 37 °C were visualized after adding Frazier’s solution [31]. Moreover, a quantitative detection of gelatinase was employed using the Bradford assay. Firstly, the Bradford protein reagent was prepared by dissolving 100 mg of Coomassie Brilliant Blue G-250 in 50 ml of 95% ethanol, then 100 ml of 85% phosphoric acid was added, and the solution was diluted to 1 L with distilled water. The amount of gelatinase in cell-free supernatant, from both treated and untreated bacteria, was determined by mixing 0.3 ml of each supernatant with 3 ml of the reagent in a test tube. After that, the absorbance at 595 nm was determined, and the percentage reduction in gelatinase enzyme production was calculated [32].

### Exopolysaccharide (EPS) production

Congo red agar plates (0.08 g of the Congo red stain in 100 ml of brain heart infusion agar and 3% sucrose) were used to determine the capability of isolates to excrete EPS. After incubation for 24 h at 37 °C, the positive isolates were identified by the black colorization of their colonies [33]. In case of EPS inhibitory effect of β-carotene measurement, a volume of 2 ml of prepared bacterial suspension (10^6^ CFU/ml) in LB broth + 1% glucose with and without β-carotene (0.5 MIC) was transferred into a 6-well, flat-bottom microplate. After 24 h of incubation at 37 °C, the non-adherent cells in treated and untreated biofilms were discarded, and 2 ml of 0.9% NaCl was added to the wells and washed thoroughly. Then, cell suspensions in 0.9% NaCl (2 ml) were transferred to sterile test tubes with an equal volume of 5% phenol. Finally, 5% v/v of concentrated sulfuric acid (2 ml) was added and incubated in the dark for 1 h, and then the absorbance was measured at 490 nm [34].

### Pyomelanin production

The pyomelanin pigmentation was identified after 48 h of incubation of the tested isolates on Muller-Hinton agar plates at 37 °C by observing a brown pigment formation [35]. The qualitative estimation of the pyomelanin inhibitory effect of β-carotene was carried out by culturing treated and untreated bacteria on Muller-Hinton agar plates at 37 °C for 48 h [35]. For quantitative determination, the P17 and P21 DTR isolates were incubated in LB broth with and without β-carotene (0.5 MIC). After shaking incubation (200 rpm) at 37 ℃ for 24 h, the bacterial culture was centrifuged, and the absorbance of the supernatant was measured at 405 nm [36].

### Rhamnolipid production

The isolates’ suspension in normal saline (0.5 McFarland) was added into the formed wells in cetyl tri ammonium bromide (CTAB)-methylene blue agar plates (methylene blue 0.02, CTAB 5, glucose 20, yeast extract 0.5, beef powder 1, peptone 10, and agar 20 g/L). After incubation for 48 h at 28 °C, the positive isolates were recognized by the formation of a blue halo around the wells (rhamnolipid producer). Where the effect of β-carotene on rhamnolipid production was determined by adding 60 µl of treated and untreated bacterial suspension to wells of CTAB-methylene blue agar plates. The formation of a blue halo was observed after incubation for 48 h at 28 °C [37].

### Siderophore production

Utilizing chrome azurol sulphonate (CAS) agar plates (10% CAS reagent in LB agar), isolates were streaked and incubated for 5 days at 28 °C. The yellow colorization of plates was observed for siderophore-producing isolates. The siderophore inhibitory effect of β-carotene was determined via streaking the overnight-incubated treated and untreated cells on CAS-agar plates, and the effect on siderophore production was determined after incubation for 5 days at 28 °C. In addition, the modified microplate method was performed for quantitative measurement; in brief, the obtained supernatant (100 µl) after centrifugation of DTR isolates in LB broth and β-carotene-containing LB broth was mixed with 100 µl CAS reagent. After 20 min, the OD at 630 nm was measured, and the siderophore was calculated in percent siderophore unit (PSU) as follows [38]:

$$\mathrm{Siderophore}\;\mathrm{production}\;(\mathrm{psu})=\frac{\mathrm{Ar}-\mathrm{As}}{\mathrm{Ar}}\times100$$

A_r_ = OD of reference (CAS solution + LB broth), and A_s_ = OD of the sample (CAS solution + supernatant of the sample).

### Hemolysin production

After streaking incubation of isolates on blood agar plates (4% human blood in LB agar) for 48 h at 28 °C, the hemolytic activity was determined by observing the formed clear zone around the tested bacteria [39]. For the qualitative assay of anti-hemolytic activity of β-carotene, the overnight-incubated treated and untreated bacteria in LB broth were spot-inoculated on human blood agar and incubated for 48 h at 28 °C [40]. For the quantitative assay, DTR isolates were inoculated in LB broth with and without 0.5 MIC β-carotene. After centrifugation, the supernatant (600 µl) was mixed with 600 µl of red blood cells (RBCs) suspension (2%) and incubated at 37 °C for 2 h. The release of hemoglobin was assessed at 540 nm after the centrifugation of the mixture for 8 min at 10,000 g (4 °C) [41]. The percentage of hemoglobin release was calculated using the following formula: [(X-B)/(T-B)] × 100], where; B is a negative control (RBCs + sterile LB broth), T is a positive control (RBCs + LB broth supplemented with 0.1% SDS), and X is the absorbance value for the sample analyzed.

### In situ visualization of antibiofilm activity of β-carotene

The microscopic examination of DTR isolates’ biofilms with and without β-carotene was carried out via light microscope (LM) and confocal laser scanning microscopy (CLSM). A bacterial suspension of 10^8^ CFU/ml was prepared in LB broth + 1% glucose with and without β-carotene (0.5 MIC), and a volume of 2 ml of the treated and untreated bacterial suspension was inoculated in 6-well microtiter plates containing 1 × 1 cm coverslips. Following incubation for 24 h at 37 °C, the glass slips were raised, washed with PBS, and stained with crystal violet (0.1%). The biofilm biomass of treated and untreated bacteria was observed by LM at 100x magnification (LABOMED, CXL, USA) [42]. In CLSM examination, the biofilms of P8, P14, and P25 DTR isolates were established in the 8-well chamber slide as mentioned above, and then the planktonic cells were eliminated by washing three times with PBS. Following 15 min of staining in the dark with 5 µl of acridine orange (green fluorescent dye), the biofilm’s structure was analyzed using CLSM (DMi8; Leica Microsystem) [43].

### Effect of β-carotene on QS genes using by quantitative reverse transcription polymerase chain reaction (qRT-PCR)

The influence of β-carotene on the expression of the QS genes, *lasR*, *rhlR*, and *pqsR* genes, was assessed by qRT-PCR. The P8, P14, and P25 DTR isolates were inoculated in 3 ml of LB broth (OD_630_ of 0.9-1.0) with 10% DMSO, as a control, and in 3 ml of LB broth with β-carotene (0.5 MIC against each isolate). Then, the broth was incubated at 37 °C for 24 h under shaking conditions. After centrifugation at 5000 rpm for 5 min at 4 °C, the supernatant was removed and bacterial cells were washed, and then the total RNA was extracted employing TRIzol reagent (Invitrogen, Carlsbad, CA, USA). After that, the complementary DNA (cDNA) was synthesized via using a cDNA synthesis kit (ThermoFisher Scientific, Waltham, MA, USA). The utilized primers for the *lasR*, *rhlR*, and *pqsR* genes, and the *rpoD* gene, a housekeeping gene, were listed in Table 1. Amplification of the selected genes was performed on cDNA of the tested isolates using a qRT-PCR device, Rotor-Gene Q (Qiagen, USA). The conditions of amplification were initial denaturation (3 min/95°C), followed by 40 cycles of denaturation (1 min/95°C), annealing (30 s/58°C), and extension (20 s/72°C). The data were analyzed using the Opticon Monitor software (version 30; Bio-Rad), and the threshold cycle (CT) value of each gene was normalized to the CT value of the *rpoD* gene, and the relative gene transcription was determined for each tested sample using the 2^-ΔΔCT^ method [44].

**Table 1:** List of primers used in qRT-PCR

Genes	Primers sequences
*rpoD*	For: GGGCGAAGAAGGAAATGGTC Rev: CAGGTGGCGTAGGTGGAGAAC
*lasR*	For: ACGCTCAAGTGGAAAATTGG Rev: GTAGATGGACGGTTCCCAGA
*rhlR*	For: AGGAATGACGGAGGCTTTTT Rev: CCCGTAGTTCTGCATCTGGT
*pqsR*	For: AACCTGGAAATCGACCTGTG Rev: TGAAATCGTCGAGCAGTACG

### In silico docking of β-carotene with LasR protein

The in silico examination was performed to study the interaction of LasR protein, the main QS regulator protein in *P. aeruginosa*, with β-carotene. At first, from the RCSB Protein Data Bank (RCSB PDB), the crystallographic structure of the LasR protein (PDB identification code: 3JPU) was obtained for the docking study in PDB format (https://www.rcsb.org/structure/3JPU). Then, the three-dimensional (3D) structure of the β-carotene ligand was downloaded in Structured Data File (SDF) format from PubChem (https://pubchem.ncbi.nlm.nih.gov/compound/5280489). The SDF format of ligand structure was converted to PDB format for the docking analysis using UCSF Chimera software. Before the beginning of docking, both LasR protein and β-carotene were energy-minimized to produce stable structures. After energy-minimization step, the preparation step was carried out by merging non-polar hydrogens and adding both polar hydrogens and Gasteiger charges for LasR protein and β-carotene using Autodock Tools (ADT) software. Following preparation of both ligand and protein, molecular analysis was achieved in many poses (*n* = 30) to predict the binding affinities of LasR protein with β-carotene. The resulting complexes between LasR and β-carotene were energy minimized using the GROMACS program, version 4.5, to verify their stability and binding analysis. From the resultant complexes between LasR and β-carotene, the most stable one was selected, its 3D structure diagram was performed using the PyMol tool, and the two-dimensional (2D) diagram was created using the LigPlot tool [45].

### In vivo model for evaluating the antipseudomonal activity of β-carotene

The antibacterial action of β-carotene against infection with DTR *P. aeruginosa* was proved using a wound infection model, and the Research Ethics Committee of the Faculty of Pharmacy at Tanta University (TP/RE/2/25 p-01) allowed the procedures in accordance with ARRIVE guidelines. The steps of the studied model were conducted as prescribed by Kamer et al. (2023) [26]. In brief, 12 male Sprague–Dawley rats weighing between 120 and 150 g were acquired from the animal house of Cairo University between the ages of 6 and 8 weeks. At first, the rats were kept separately in vented cages with 12 h of light and 12 h of darkness, at room temperature, and with unhindered access to food and drink to give them time to adjust to their surrounding environment. Before the creation of wounds, the rats had been anesthetized with ketamine (40 mg/kg) and xylazine (5 mg/kg), and their backsides were shaved. After that, the shaved areas were sterilized with 10% povidone-iodine, and the excisional wounds (10 mm in diameter) were performed by the aid of biopsy punches. After wound creation, each rat was kept in a separate cage to avoid fighting and contamination between them. A volume of (10 μl) of bacterial suspension of P25 isolate (106 CFU/ml) in normal saline was subcutaneously injected into the developed wounds in all rats, then the rats were divided into 2 groups (control group and treated group), each group comprised of 6 rats. The control group (group 1) was subcutaneously administered 20 μl of 10% DMSO as a negative control, whereas the treated group (group 2) was subcutaneously injected with 20 μl of β-carotene (25 μg/ml; MIC against P25 isolate) on days 0, 3, and 6. The wound diameter was measured using a sterile ruler on days 0, 3, and 6, and the percentages of wound contractions were calculated using the formula below.

On the sixth day of the experiment, rats from each group were put to death by CO₂ inhalation, and the skin lesions were excised (2–5 mm of the area). The histological analysis of the skin using hematoxylin and eosin (H & E) was conducted at the pathology department at the Faculty of Medicine, Tanta University. In addition, the skin tissues from each group in PBS were homogenized using a sonicator, and the resulting suspensions were serially diluted (dilution factor = 10), and 1 ml from each dilution was inoculated into a sterile petri dish (10 cm), and then a volume of 19 ml of molten Cetrimide agar was added to each plate. Finally, the plates were shaken to equally distribute the bacterial suspension and incubated at 37 °C for 24 h. The formed CFU on Cetrimide agar were counted, and bacterial burdens in skin tissue from both the control and treated groups were counted using the following formula:

$$\mathrm{CFU}/\mathrm g=\left(\mathrm{plate}\;\mathrm{count}\times\frac1{\mathrm{dilution}}\times10\right)/\mathrm{weight}\;\mathrm{of}\;\mathrm{homogenized}\;\mathrm{tissue}$$

### Statistical analysis

The data were reported as mean ± standard deviation (SD), and the experiments were run in triplicate. GraphPad Prism version 5 software was utilized to compare the two groups using a *t*-test in case of normally distributed data. In the case of non-normally distributed data, the nonparametric Mann-Whitney test was conducted. The normality test was conducted using the Shapiro-Wilk test, and a p-value < 0.05 served as a significance level.

## Results

### Bacterial isolates

In our study, a total of 100 isolates from different sources (sputum (50), urine (30), wound (16), and blood (4)) were identified as *P. aeruginosa* based on their morphological features on Cetrimide agar plates and their biochemical test results (positive for motility, citrate, and oxidase tests, and negative for urease, indole, methyl red, and Kligler’s iron agar tests).

### Antimicrobial resistance (AMR) and resistotyping of MDR/DTR isolates

The percentage of AMR of the tested *P. aeruginosa* (*n* = 100) against ciprofloxacin, levofloxacin, meropenem, imipenem–cilastatin, cefepime, ceftazidime, gentamycin, aztreonam, polymyxin B, piperacillin/tazobactam, tobramycin, and colistin were 60%, 54%, 32%, 38%, 24%, 22%, 29%, 81%, 0%, 60%, 15%, and 0%, respectively. Among the tested bacteria, it was found that 31 isolates were MDR and 9 isolates were DTR. The tested MDR/DTR isolates (*n* = 40) displayed the highest percentages of resistance against ciprofloxacin (95%), levofloxacin (82.5%), and aztreonam (80%). Controversy, there was no isolate resistant to polymyxin B (0%) and colistin (0%). The percentages of AMR of MDR/DTR isolates against the remaining antibiotics ranged from 35 to 70%. The distribution of the various resistotypes detected between the tested MDR/DTR *P. aeruginosa* isolates and the percentages of AMR are presented in Table 2.

**Table 2:**
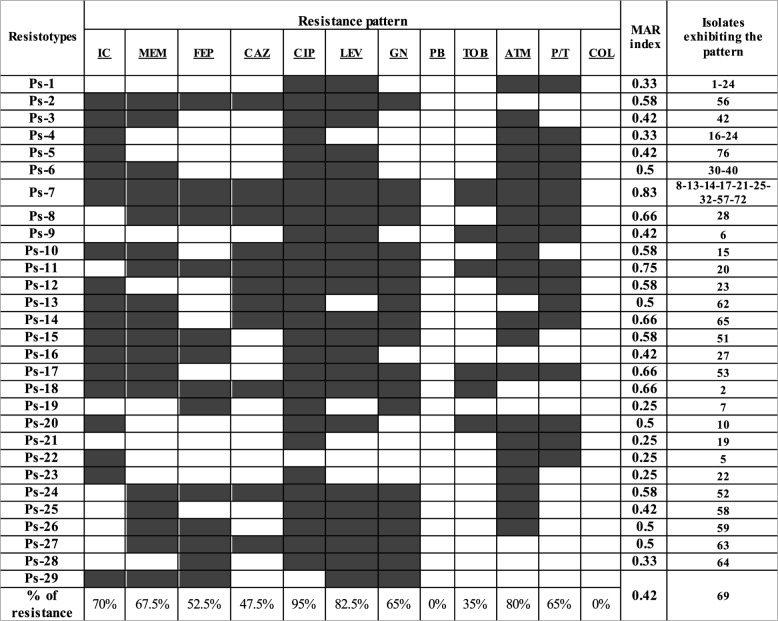
Resistance pattern of MDR/DTR *P. aeruginosa* isolates (*n* = 40)

The grey areas in the resistance pattern refer to resistance to antibiotics, and the white areas in the resistance pattern refer to sensitivity to antibiotics. *IC* imipenem–cilastatin, *MEM* meropenem, *FEP* cefepime, *CAZ* ceftazidime, *CIP* ciprofloxacin, *LEV* levofloxacin, *GN* gentamicin, *PB* polymyxin B, *TOB* tobramycin, *ATM* aztreonam, *P/T* piperacillin/tazobactam, *COL* colistin

### Antipseudomonal activity and MIC of β-carotene against MDR/DTR *P. aeruginosa* isolates

**Fig. 1:**
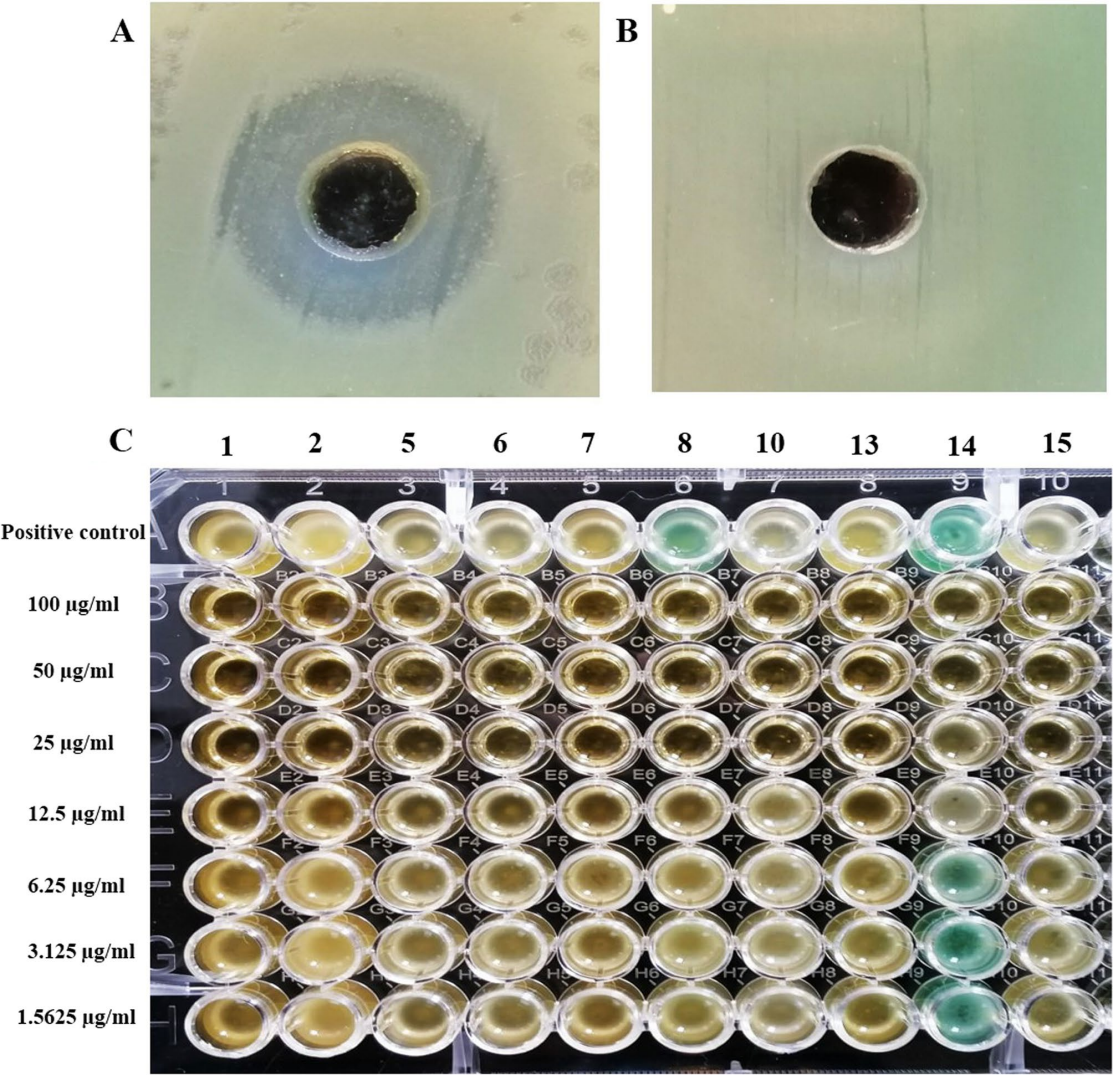
Antipseudomonal action of β-carotene against MDR/DTR *P. aeruginosa*. **A** A representative inhibition zone was produced by β-carotene (200 µg/ml); **B** A negative control (10% DMSO) showed no inhibition zone. **C**. A representative microtiter plate showing the MIC values of β-carotene against MDR/DTR *P. aeruginosa*

Antipseudomonal activity of β-carotene (200 µg/ml) was inspected using the well-diffusion method against MDR/DTR isolates (*n* = 40), and the measured inhibition zones ranged from 10 to 33 mm, where the observed MICs using the microdilution approach ranged from 12.5 to 100 µg/ml, as displayed in Fig. 1; Table 3. The negative control (10% DMSO) showed no inhibition zone against all tested isolates.

**Table 3:** The measured inhibition zones and MIC of β-carotene against MDR/DTR *P. aeruginosa*

**MDR strains **	**Inhibition zone (mm)**	**MIC** **(µg/ml)**	**MDR strains**	**Inhibition zone (mm)**	**MIC** **(µg/ml)**
**P1**	25	25	**P28**	33	25
**P2**	22	25	**P30**	30	25
**P5**	20	25	**P32**	21	25
**P6**	22	25	**P40**	21	25
**P7**	30	25	**P42**	24	25
**P8**	19	25	**P51**	29	25
**P10**	20	25	**P52**	20	50
**P13**	28	25	**P53**	23	25
**P14**	17	50	**P56**	17	50
**P15**	30	25	**P57**	14	50
**P16**	25	25	**P58**	13	50
**P17**	22	25	**P59**	18	50
**P19**	32	25	**P62**	32	25
**P20**	30	12.5	**P63**	22	25
**P21**	17	25	**P64**	25	25
**P22**	17	50	**P65**	12	50
**P23**	17	50	**P69**	10	100
**P24**	21	25	**P72**	25	25
**P25**	30	25	**P76**	32	25
**P27**	22	25	**P28**	30	25

### Virulence factors scoring of MDR/DTR P. aeruginosa isolates

A total of 10 virulence factors, namely biofilm, pyocyanin, motility, protease, gelatinase, EPS, pyomelanin, rhamnolipid, siderophore, and hemolysin, were screened in MDR/DTR *P. aeruginosa* isolates. The virulence score (VS) for each isolate was calculated, and the most virulent isolates were selected for subsequent study. From Table 4, it was observed that the DTR isolates (P8, P13, P14, P17, P21, P25, P32, P57, and P72) were the most virulent *P. aeruginosa* isolates with VS = 15.

**Table 4:** The VS of MDR/DTR *P. aeruginosa* isolates (*n* = 40)

MDR strains	A		B		C		D	E	F	G	H	I	L	VS
P1	0.184	+	0.313	++	19	+	-	+	-	-	-	+	-	6
P2	0.23	++	0.33	++	18	+	-	-	-	-	-	-	+	6
P5	0.153	+	0.133	+	12	+	-	-	-	-	-	-	+	4
P6	0.167	+	0.167	+	12	+	-	-	-	-	-	-	+	4
P7	0.116	+	0.021	+	12	+	-	-	-	-	-	-	+	4
**P8**	0.822	+++	0.949	+++	33	+++	+	+	+	-	+	+	+	**15**
P10	0.333	++	0.229	++	20	++	-	-	+	-	-	-	-	7
**P13**	0.637	+++	0.878	+++	38	+++	+	+	+	-	+	+	+	**15**
**P14**	0.87	+++	1.261	+++	35	+++	+	+	+	-	+	+	+	**15**
P15	0.184	+	0.26	++	12	+	-	-	-	-	+	+	+	7
P16	0.248	++	0.339	++	18	+	-	-	-	-	-	-	-	5
**P17**	0.597	+++	0.793	+++	29	++	+	+	+	+	+	+	+	**15**
P19	0.31	++	0.312	++	18	+	-	-	+	-	-	-	+	7
P20	0.206	++	0.368	++	16	+	-	-	-	-	-	-	+	6
**P21**	0.65	+++	0.713	+++	26	++	+	+	+	+	+	+	+	**15**
P22	0.257	++	0.39	++	16	+	-	-	-	-	-	+	+	7
P23	0.373	+++	0.338	++	18	+	-	-	+	-	-	-	+	8
P24	0.147	++	0.122	+	12	+	-	-	-	-	-	-	+	5
**P25**	0.747	+++	0.993	+++	35	+++	+	+	+	-	+	+	+	**15**
P27	0.229	++	0.361	++	18	+	-	-	-	-	-	-	-	5
P28	0.143	+	0.179	+	12	+	-	-	-	-	-	-	+	4
P30	0.376	++	0.205	++	13	+	-	-	+	-	-	-	+	7
**P32**	0.71	+++	1.293	+++	35	+++	+	+	+	-	+	+	+	**15**
P40	0.142	+	0.287	++	18	+	-	-	-	-	-	+	-	5
P42	0.13	+	0.094	+	12	+	-	-	-	-	-	-	-	3
P51	0.252	++	0.492	+++	18	+	-	-	-	-	-	-	-	6
P52	0.147	+	0.367	++	16	+	-	-	-	-	-	-	+	5
P53	0.28	++	0.303	++	18	+	-	-	-	-	-	-	+	6
P56	0.197	+	0.024	+	19	+	-	-	-	-	-	-	+	4
**P57**	0.79	+++	1.43	+++	36	+++	+	+	+	-	+	+	+	**15**
P58	0.116	+	0.218	++	18	+	-	-	-	-	-	-	+	5
P59	0.257	++	0.225	++	17	+	+	-	-	-	-	-	+	7
P62	0.32	++	0.046	+	12	+	-	-	+	-	-	-	+	6
P63	0.192	+	0.008	+	12	+	-	-	-	-	-	-	+	4
P64	0.22	++	0.318	++	16	+	-	-	-	-	-	-	+	6
P65	0.27	++	0.039	+	18	+	-	-	-	-	-	-	+	5
P69	0.285	++	0.332	++	19	+	-	-	-	-	-	+	-	6
**P72**	0.682	+++	1.119	+++	34	+++	+	+	+	-	+	+	+	**15**
P76	0.277	++	0.392	++	18	+	-	-	-	-	-	-	+	6

A; biofilm (+ >0.2, 0.2 ≥ ++ ≥ 0.4, and **+++** <0.4), B; pyocyanin (+ >0.2, 0.2 ≥ ++ ≥ 0.4, and **+++** <0.4), C: motility (+ >20 mm, 20 mm ≥ ++ ≥ 30 mm, and **+++** <30 mm), D; protease, E; gelatinase, F; EPS, G; pyomelanin, H; rhamnolipid, I; siderophore, and L; hemolysin. + indicates a positive result, and – indicates a negative result.

### Growth curve analysis of DTR *P. aeruginosa* isolates

**Fig. 2:**
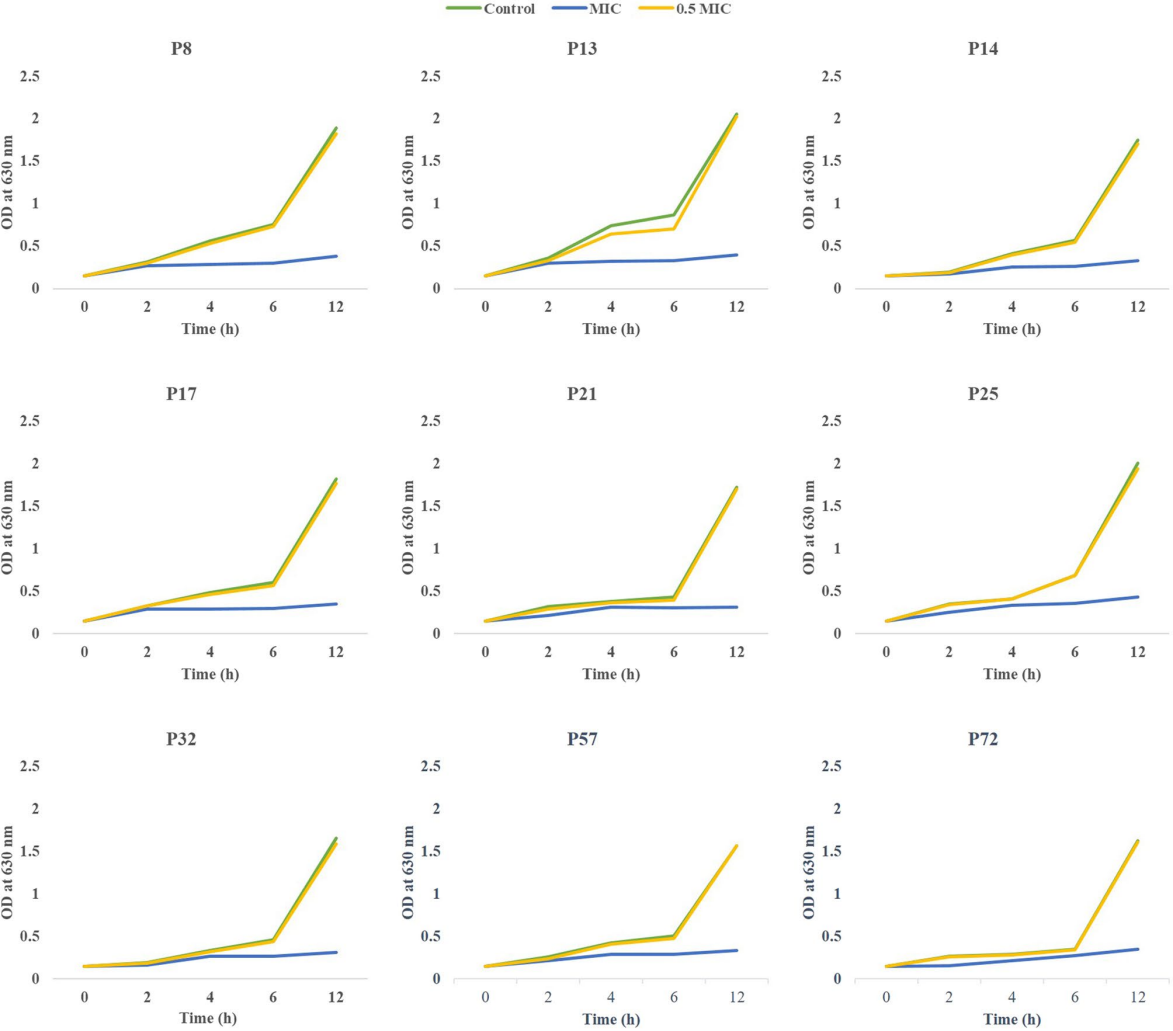
Growth curves of DTR *P. aeruginosa* isolates (*n* = 9) after treatment with 0, 0.5, and 1 MICs of β-carotene

The impact of 0, 0.5, and 1 MICs of β-carotene against the highly virulent DTR *P. aeruginosa* isolates was investigated at 0 h, 2 h, 4 h, 6 h, and 12 h by measuring OD at 630 nm. The OD measurements of MIC-treated isolates showed that treating with the MIC of β-carotene considerably affected the growth curve in all tested isolates. On the other hand, the OD measurements of 0.5 MIC-treated isolates revealed that treatment with 0.5 MIC of β-carotene had approximately no effect on the growth in all tested isolates, manifesting the same curves as the control ones (Fig. 2).

### In vitro virulence attenuation of DTR *P. aeruginosa* isolates via 0.5 MIC of β-carotene

**Fig. 3:**
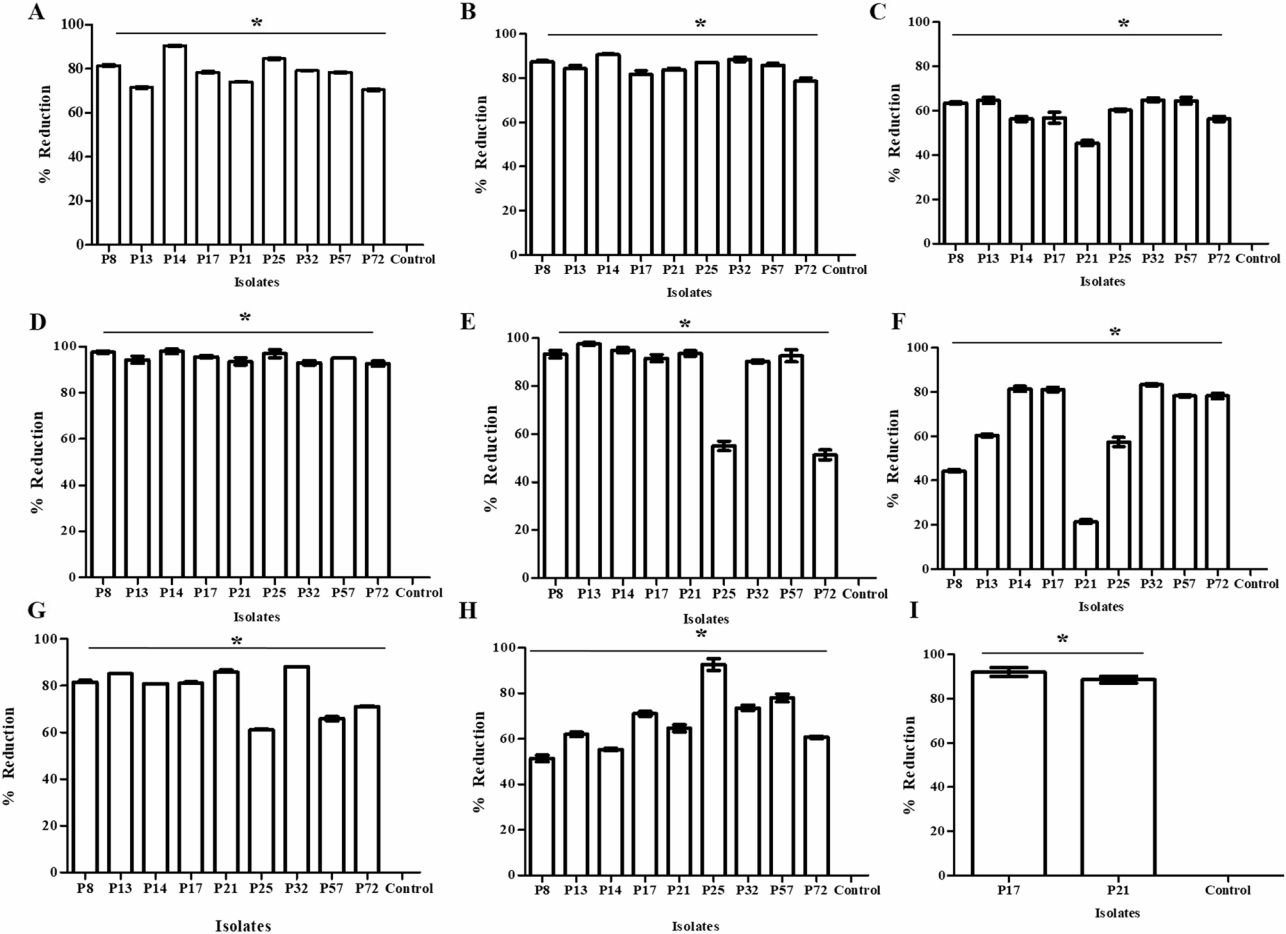
Antivirulence activity of β-carotene (0.5 MIC) against DTR isolates. **A** The percentage of reduction in biofilm formation. **B** The percentage of reduction in pyocyanin production. **C** The percentages of reduction in motility. **D** The percentage of reduction in protease production. **E** The percentage of reduction in gelatinase production. **G** The percentage of reduction in EPS production (**F**). The percentage of reduction in siderophore production. **H** The percentage of reduction in hemolysin production. **I** The percentage of reduction in pyomelanin production. Each bar represents the mean of three replicates, and the error bar represents the SD. The asterisk signifies significance at *P* < 0.05 (Mann-Whitney test)

**Fig. 4:**
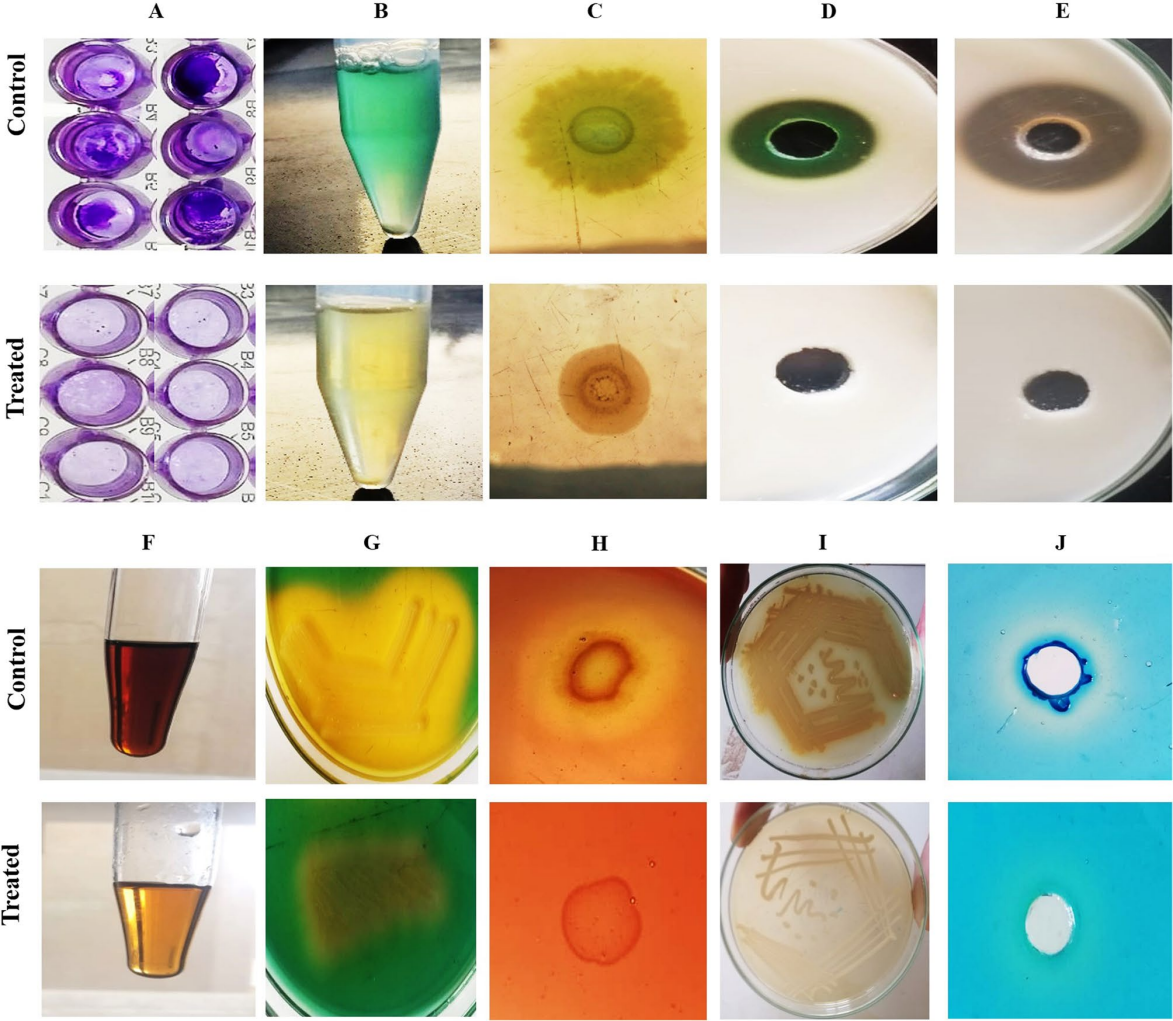
Representative images of the antivirulent action of β-carotene (0.5 MIC) against the P17 DTR isolate. **A** Wells of microtitre plates displayed a reduction in biofilm biomass (violet stain) after treatment with β-carotene. **B** Loss of pyocyanin pigmentation (green color) of broth medium after adding β-carotene. **C** The motility diameter of the P17 isolate was reduced via treatment with β-carotene. **D** Casein agar plates indicated the loss of protease activity after adding β-carotene, and the loss of a clear zone around the well. **E** Gelatin agar plates showed the loss of gelatinase activity after adding β-carotene, and loss of the clear zone around the well. **F** Phenol-sulfuric acid test manifested a reduction in the production of EPS in the P17 isolate after treatment with β-carotene, lessening the amplitude of color from dark red to yellow. **G** The CAS-agar plate displayed the loss of yellow colorization due to siderophore production in the treated isolate. **H** The blood agar plate showed the complete loss of hemolytic activity in the treated isolate, and loss of the transparent zone formed around the bacterial growth. **I** Muller-Hinton agar plate showed the loss of brown pigmentation (pyomelanin pigment) in the treated isolate. **J** The CTAB-agar plate showed the disappearance of the dark blue halo of rhamnolipid in the treated isolate

After screening of the antivirulence activity of β-carotene (0.5 MIC) against the most virulent DTR *P. aeruginosa*, it was reported that β-carotene significantly (*P* > 0.05) diminished the biofilm formation by 70.4–90.5%, and the amplitude of crystal violet stain was lower in treated wells compared to untreated wells. The intensity of green colorization of broth due to pyocyanin production decreased in treated tubes when compared to untreated tubes, and the percentages of reduction ranged from 78.6 to 90.7%. The tryptone swimming plates displayed motility zones with smaller diameters in treated bacteria compared to untreated ones, with inhibitory percentages ranging from 45.4 to 64.9%. The formed clear zones in skim milk agar plates were larger for untreated supernatants in comparison with treated supernatants, and the protease activity inhibited by 100% for all tested isolates. As well, the quantitative assay using Folin-Ciocalteu reagent displayed that the production of protease reduced by 92.6–98% after treatment with 0.5 MIC of β-carotene. The resultant zones after adding Frazier’s solution in gelatine agar plates manifested that 0.5 MIC of β-carotene repressed the production of gelatinase enzyme by 43.9–100%. Additionally, the quantitative assay using Bradford method showed that the gelatinase production inhibited by 51.3–97.6% after treatment with 0.5 MIC of β-carotene. Phenol sulfuric acid assays of EPS in treated and untreated isolates unveiled the inhibitory effect of β-carotene on EPS production, showing an inferior red colorization after treatment with β-carotene, and the percentages of suppression ranged from 21.5 to 83.3%. The CAS agar plates showed that the yellow colorization of plates due to siderophore production disappeared with β-carotene application (0.5 MIC), with percentages of inhibition ranging from 61 to 88%. The hemolytic activity of all tested isolates on blood agar plates was inhibited by treating with 0.5 MIC of β-carotene, and the percentages of inhibition ranged from 51.4 to 92.6%. The production of pyomelanin pigment by P17 and P21 was repressed after treatment with 0.5 MIC of β-carotene, and the percentages of inhibition were 88.6% and 92%, respectively. The CTAB agar plates manifested the prevention of rhamnolipid production in all tested bacteria, lacking blue halo formation, after treatment with 0.5 MIC of β-carotene (Figs. 3 and 4).

### In situ examination of antibiofilm activity of β-carotene against DTR *P. aeruginosa* isolates using LM and CLSM

**Fig. 5:**
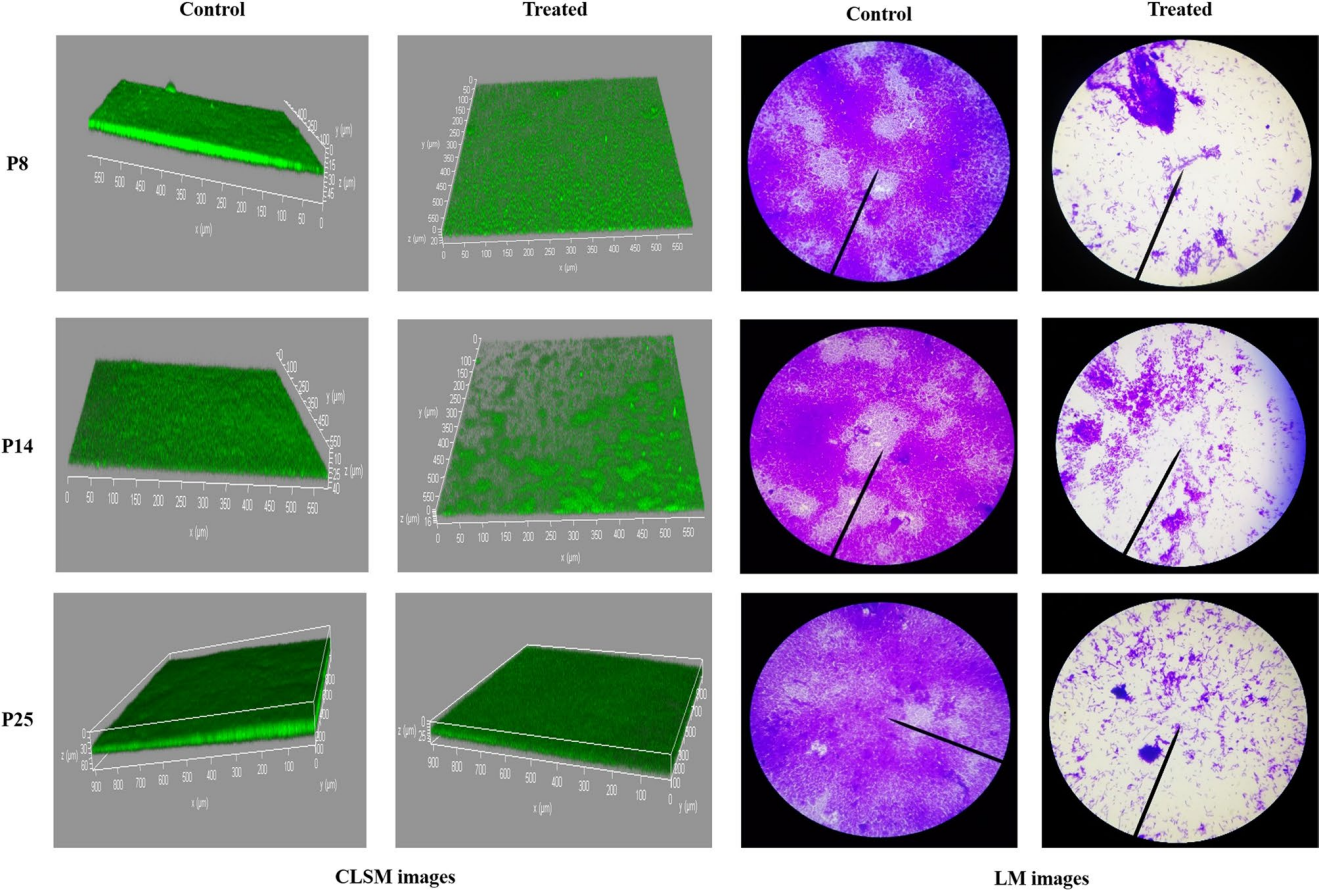
In situ examination of the antibiofilm activity of P8, P14, and P25 DTR isolates. Images of LM manifested the inhibition in biofilm formation of DTR *P. aeruginosa* isolates after treatment with 0.5 MIC of β-carotene. Images of CLSM displayed a reduction in biofilm biomass after treatment with 0.5 MIC of β-carotene, while the untreated DTR isolates showed thick, mature biofilms

The effect of β-carotene on biofilm formation was further studied by microscopic examinations. After examination of the formed biofilm of DTR isolates P8, P14, and P25 with and without β-carotene treatment (0.5 MIC) via LM, it was revealed that biofilm formation in all isolates was significantly inhibited. As shown in Fig. 5, the biofilm biomass decreased after treatment with β-carotene in all tested bacteria. On the other hand, the untreated bacteria formed well-established biofilms with massive biomass. Furthermore, CLSM for DTR isolates P8, P14, and P25 confirmed the considerable reduction in biofilm biomass after treatment with β-carotene, and the thickness of P8, P14, and P25 biofilms was significantly reduced from 45 μm to 20 μm, 40 μm to 16 μm, and 60 μm to 25 μm, respectively (Fig. 5).

### Downregulation of QS genes in DTR *P. aeruginosa* isolates via 0.5 MIC of β-carotene

**Fig. 6:**
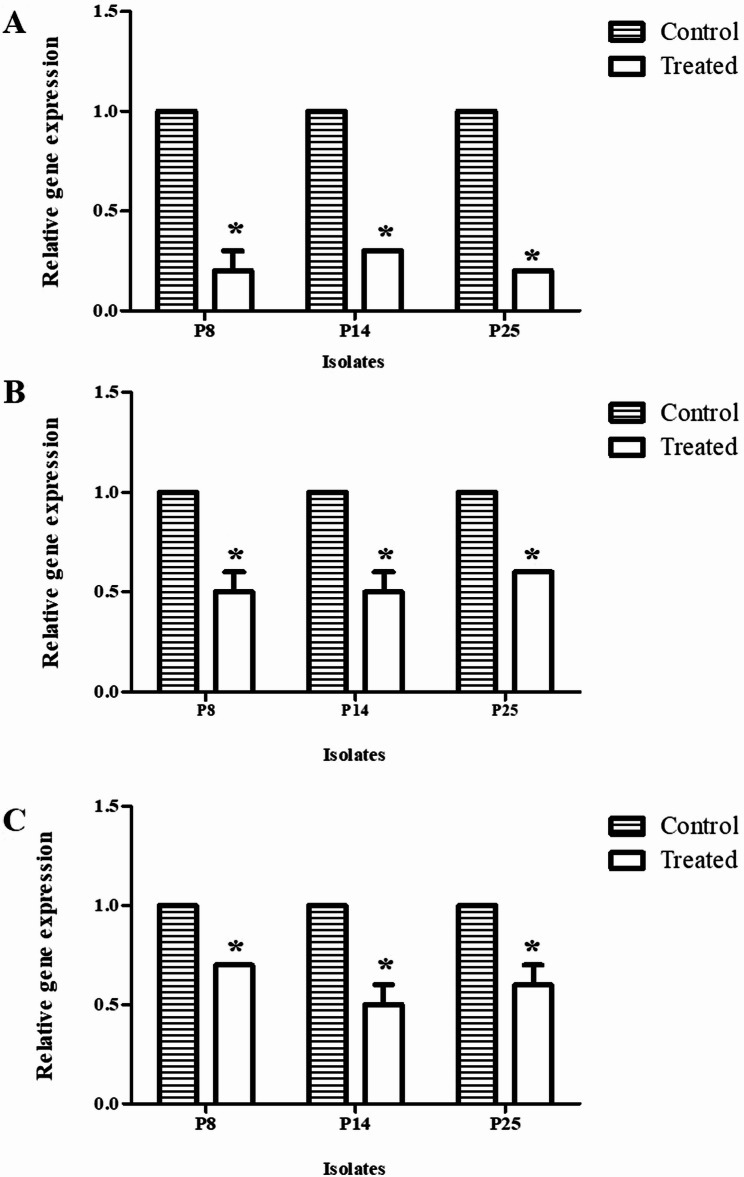
Effect of β-carotene on the relative gene expression of quorum sensing genes in P8, P14, and P25 MDR isolates. **A** The expression of *lasR* gene. **B** The expression of *rhlR* gene. **C** The expression of *pqsR* gene. Each bar represents the mean of three replicates; the error bar represents the SD; asterisk signifies significance at *P* < 0.05 (*t*-test)

The anti-QS action of β-carotene was investigated by examining the effect of 0.5 MIC of β-carotene on QS genes, namely *lasR*, *rhlR*, and *pqsR* genes, on P8, P14, and P25 DTR isolates via qRT-PCR. The data revealed that β-carotene significantly (*P* < 0.05) reduced the expression of the examined genes in all tested isolates, compared to the negative control (10% DMSO) that had no effect on the expression of these genes (relative gene expression for all tested genes = 1). In contrast, the relative gene expressions of the *lasR* gene after β-carotene treatment were 0.2, 0.3, and 0.2 in P8, P14, and P25 isolates, respectively. The relative gene expressions of the *rhlR* gene were 0.5, 0.5, and 0.6 in β-carotene-treated P8, P14, and P25 isolates, respectively. For the *pqsR* gene, its relative expressions in P8, P14, and P25 isolates were 0.7, 0.5, and 0.6, respectively. Additionally, the data showed that the most affected QS gene was *lasR*, followed by *rhlR* and *pqsR* genes, as shown in Fig. 6.

### Blocking of QS by LasR-β-carotene complex formation

**Fig. 7:**
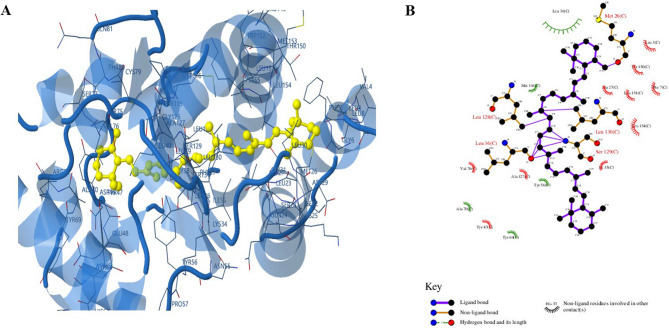
Molecular docking showing LasR-β-carotene complex formation. **A** The 3D structure revealed the fitting of β-carotene (yellow color) in the active site of the LasR protein. **B** The 2D structure showed the interacting amino acids with β-carotene

In addition to studying the effect of β-carotene on QS genes, the anti-QS was also examined by investigating the effect of β-carotene on the key regulator of the QS system, the LasR protein. LasR protein acts as a global transcriptional activator since it controls the expression of various virulence factor-associated genes. After preparation of the LasR protein, 3D protonation, and docking, it was found that the β-carotene ligand interacted with the LasR protein by making a hydrophobic bond with methionine (Met) 26, leucine (Leu) 36, Leu 128, serine (Ser) 129, and Leu 130 amino acids with a binding free energy of −8.6 kcal/mol (Fig. 7). This strong bond between β-carotene and LasR protein blocked its regulatory role in QS, leading to a lowering of the expression of QS genes and the production of virulence factors.

### β-Carotene induced wound healing in DTR *P. aeruginosa****-***infected rats

**Fig. 8:**
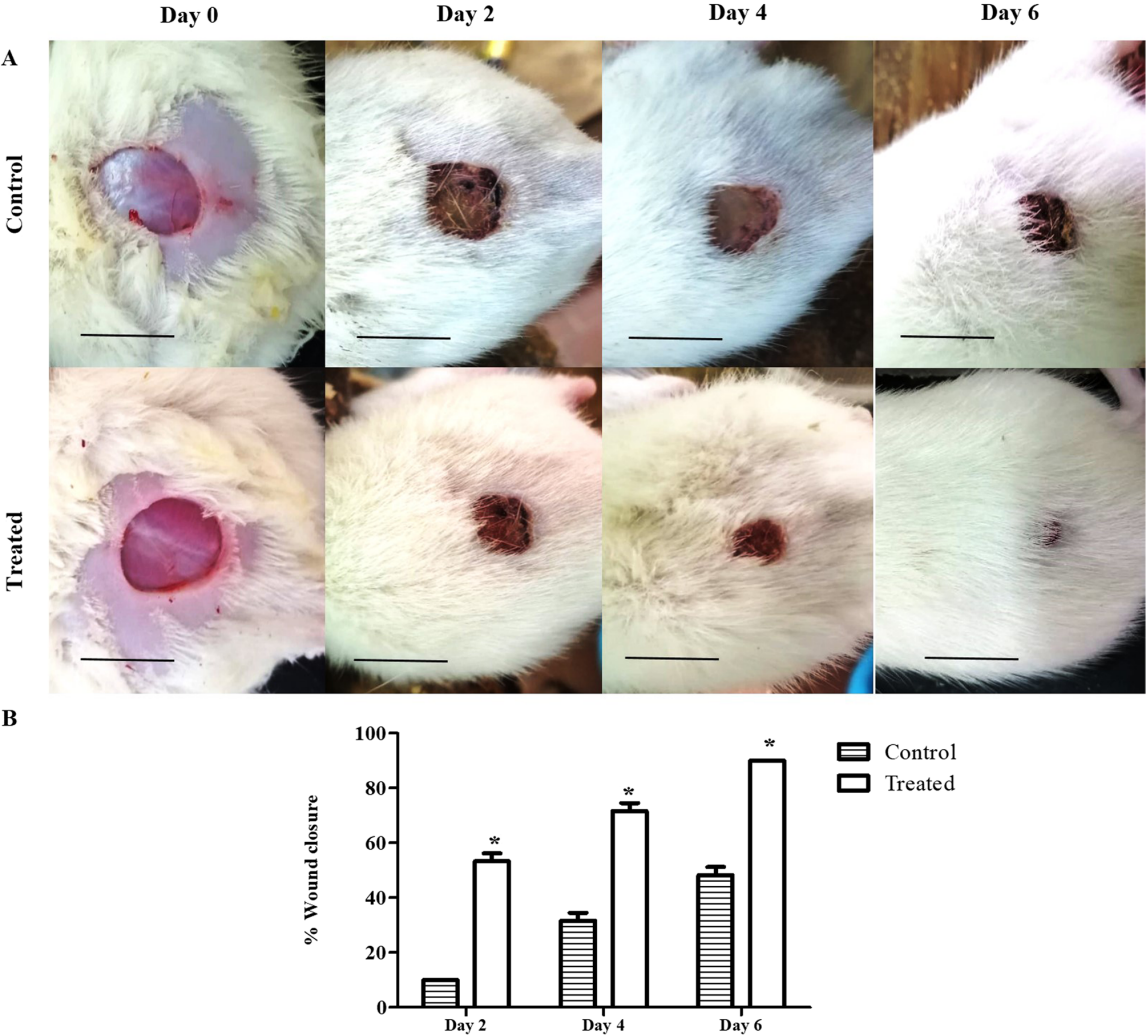
β-carotene treatment ameliorated wound closure in P25-infected rats. **A** Representative images revealed the wound closure with days in control and treated groups (n per group = 6). **B** The bar chart represents the percentage of wound closure in the control and treated groups. Each bar represents the mean of three replicates, and the error bar represents the SD. The asterisk signifies significance at *P* < 0.05 (*t*-test). Scale bar = 10 mm

The wound infection model was used to assess the ability of β-carotene to mitigate infection by *P. aeruginosa* (DTR P25 isolate). The wound closure was measured every 2 successive days in the control and treated (25 µg/ml) groups. As presented in Fig. 8, β-carotene treatment consistently accelerated wound closure, and the percentages of wound closure were significantly (*P <* 0.05) higher in treated rats compared to untreated rats on different days of the experiment.

### β-Carotene lowered *Pseudomonas* burden and improved skin histopathological features

**Fig. 9:**
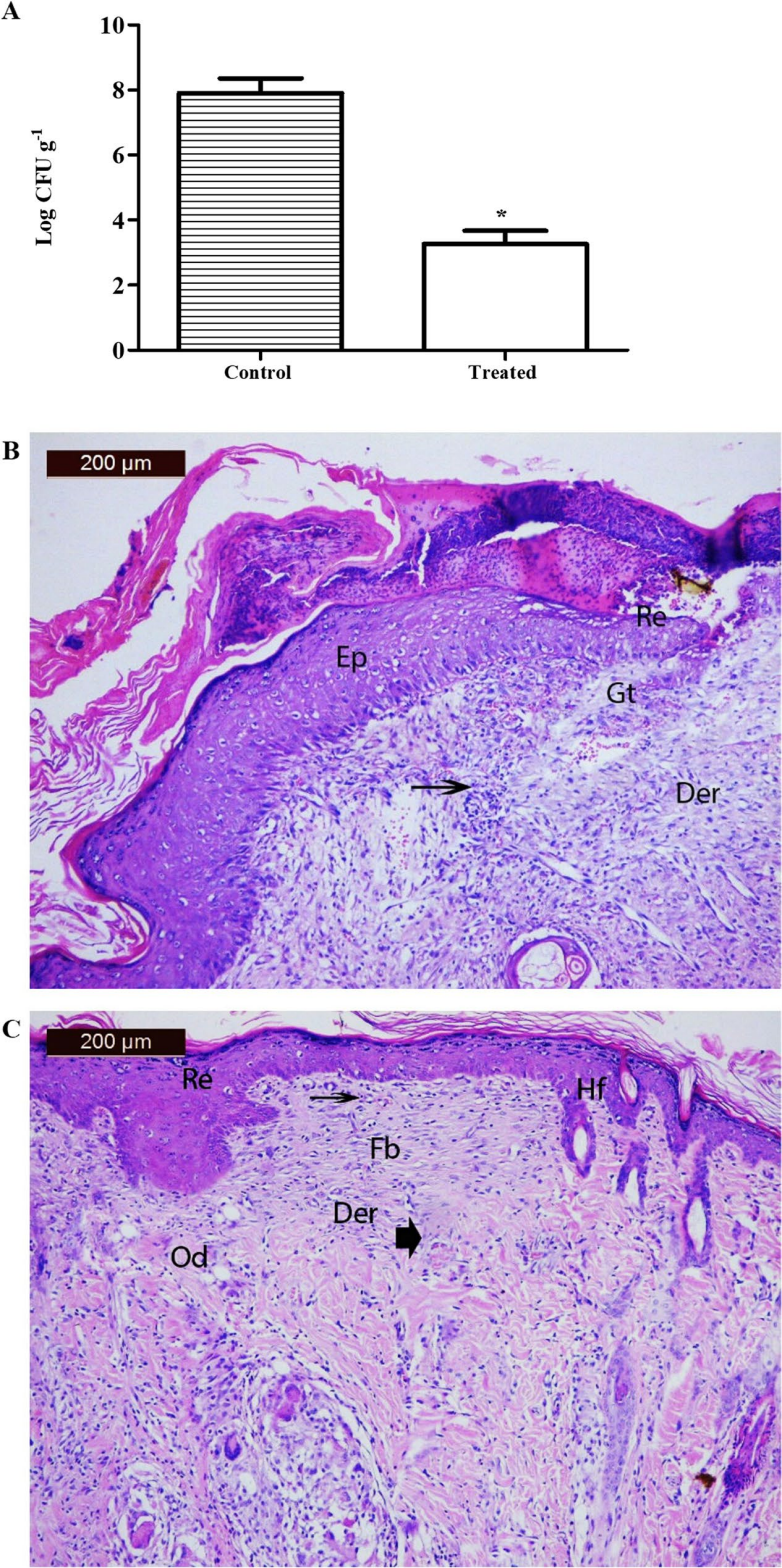
**A** *Pseudomonas* burdens in control and treated groups expressed by mean log CFU/g. **B** Photomicrograph of a section in rat skin from the control group showed a discontinuous, thickened epidermis (Ep) with underlying granulation tissues (Gt) containing sub-epidermal necrotic dermis (Der), and loss of hair follicles and sebaceous glands. Note mild re-epithelialization (Re) of the epidermal layer overlaid by scales with a small crust containing inflammatory cells (arrow) that adhere to the epithelium at the edge of the unhealed wound surface. **C** Photomicrograph of a section in rat skin from the wound + treatment group showing re-epithelialization (Re) in the epidermis layer, mild discontinuous epidermis (Ep), and mild necrosis in the dermis (Der), an inflammatory infiltrate of cells (arrow), and an early granulation tissue composed of activated fibroblasts (Fb), with few capillaries (arrowhead) and newly formed hair follicles (HF). The asterisk signifies significance at *P* < 0.05 (*t*-test)

The *Pseudomonas* burdens in both control and β-carotene-treated groups were determined by measuring CFU/g of homogenized tissue from infected wounds. As displayed in Fig. 9A, the mean log CFU/g of *Pseudomonas* burden in the control infected group equals 7.9; on the other hand, the mean log CFU/g of *Pseudomonas* burden in the treated infected group equals 3.3 (up to 58% reduction in *Pseudomonas* burden). Histopathological examination of the skin of the control group showed discontinuous thickened epidermis with underlying granulation tissues containing sub-epidermal necrosis, loss of hair follicles, and sebaceous glands. Note mild re-epithelialization of the epidermal layer overlaid by scales with a small crust containing inflammatory cells adhering to the epithelium at the edge of the unhealed wound surface (Fig. 9B). In contrast, the examination skin sections from the treated group showed re-epithelialization in the epidermis layer, mild discontinuous epidermis, and mild necrosis in the dermis, an inflammatory infiltrate of cells, and early granulation tissue composed of activated fibroblasts, a few capillaries, and newly formed hair follicles (Fig. 9C). Overall, our data indicated that β-carotene played a positive role in the healing process of the DTR *P. aeruginosa*-infected wounds, and the healing mechanisms were related to its antibacterial action as well as its inhibitory effect on the production of virulence factors via downregulating the expression of virulence genes and blocking the QS-regulatory effect of LasR protein.

## Discussion

The AMR bacteria-caused infectious diseases represent a serious threat to healthcare systems; in 2019, bacterial AMR was estimated to be responsible for 4.95 million deaths worldwide, including 1.27 million deaths directly related to bacterial AMR [46]. *P. aeruginosa* is one of the six main bacterial pathogens that cause AMR-related deaths [47]. The term “difficult-to-treat resistance” was first used in 2018, when *P. aeruginosa* demonstrated non-susceptibility to all of the following antimicrobial agents: aztreonam, imipenem-cilastatin, ciprofloxacin, levofloxacin, ceftazidime, cefepime, piperacillin-tazobactam, and meropenem [48]. The management of DTR *P. aeruginosa*-associated infections signifies a considerable challenge [3]. When it comes to resistant pathogens, especially carbapenem-resistant Enterobacteriaceae, polymyxins are frequently the only effective antibiotic agent [49]. However, the multiple dose-dependent side effects of polymyxins, including renal impairment, neurotoxicity, and respiratory depression, limit their utility in medical settings [50]. Therefore, the existing study focused on investigating a new treatment option for DTR *P. aeruginosa* by evaluating the antibacterial, antivirulence, and anti-QS activities of β-carotene, a safe carotenoid pigment (no negative effects were observed at β-carotene dose = 500 mg/kg) [51], against the highly virulent DTR *P. aeruginosa*.

Our data revealed for the first time that the β-carotene exhibited a profound antipseudomonal activity against all tested MDR *P. aeruginosa*, including DTR isolates, at a concentration of 200 µg/ml. The antibacterial properties of carotenoids have been documented in numerous studies against various bacterial species [52]. The study conducted by Kusmita et al. (2023) showed that the carotenoid exerted antibacterial activity against methicillin-resistant *S. aureus* (MRSA) and MDR *Escherichia coli* [53]. In addition, Hagaggi & Abdul-Raouf (2023) reported that the β-carotene exhibited promising antibacterial activity against *S. aureus*, *S. agalactiae*, *P. aeruginosa*, and *K. pneumoniae* at a concentration of 1 mg/ml [16].

Biofilm bacteria are more resistant to antibiotic doses than planktonic cells; therefore, it is more challenging to remove a chronic disease associated with biofilm formation [54]. Thus, the effect of β-carotene against biofilm formation by DTR *P. aeruginosa* was evaluated in the current study. For the first time, our study revealed that β-carotene had a significant influence on the inhibition of biofilm formation by DTR *P. aeruginosa* (up to 90.5%), which was confirmed via LM and CLSM that showed a dramatic reduction in biofilm biomass. In agreement with our findings, Majeed (2017) reported the antibiofilm activity of carotenoids against *E. coli*, *Bacillus subtilis*, *S. aureus*, *K. pneumoniae*, and *P. aeruginosa* at a dose of 1 mg/ml [55]. In addition, *P. aeruginosa*’s biofilm was reduced by 50% in the presence of 2–4 µg/ml of the *Rhodococcus* sp. SC1’s carotenoid pigment [56].

Additionally, the infection by biofilm-forming bacteria becomes more serious along with the production of virulence traits, such as protease, pyocyanin, and motility [57]. Our produced β-carotene also showed an intensive reduction in all tested virulence factors of DTR *P. aeruginosa*, including pyocyanin (up to 90.7%), motility (up to 64.9%), protease (up to 100%), gelatinase (up to 100%), and EPS (up to 83.3%). Additionally, the production of pyomelanin, rhamnolipid, siderophore, and hemolysin was inhibited after treatment with 0.5 MIC of β-carotene. According to Sampathkumar et al. (2019), when *P. aeruginosa* was treated with lutein, a carotenoid pigment, their biofilm formation, pyocyanin production, cell surface hydrophobicity, and extracellular polymeric substances were disrupted [58].

Moreover, bacteria frequently communicate with one another to regulate the creation of biofilms and the production of pathogenicity through a process known as QS. Stopping the creation of biofilm and virulence through blocking QS may prevent the spread of disease [59, 60]. Hence, the influence of β-carotene against QS was examined in our study genotypically and by molecular analysis. It was found that the expression of all tested genes, including *lasR*, *rhlR*, and *pqsR* genes, was significantly suppressed after treatment with 0.5 MIC of β-carotene, and the most affected gene was the *lasR* gene (80% suppression). Where the in silico analysis displayed a strong bond formation between β-carotene and LasR protein, the main QS regulator, which in turn downregulated the virulence factor-associated genes and lessened the production of all the examined virulence factors of *P. aeruginosa* as stated above (21.5–100% inhibition). Many previous studies have discussed the role of blocking of LasR protein in disarming QS and virulence of *P. aeruginosa*. Soltane et al. (2023) reported that norlobaridone specifically bound and blocked *P. aeruginosa*’s LasR protein, which in turn reduced the formation of *P. aeruginosa* biofilms (64.6% inhibition) and their associated virulence factors, including pyocyanin and rhamnolipids (% inhibition = 61.1% and 55%, respectively) [61]. In another study, bakuchiol was regarded as a strong QS inhibitor because it destabilized LasR protein upon binding and interfered with its function, suppressing the formation of *P. aeruginosa* biofilms (75.5% inhibition) and the virulence factors that are linked to them, such as pyocyanin and rhamnolipids (71.5% and 66.9% inhibition, respectively) [62]. Moreover, Alhadrami et al. (2025) found that the optimized peptide inhibitor Aqs1C efficiently repressed QS-associated virulence factors in *P. aeruginosa*, involving biofilm formation, pyocyanin production, protease secretion, and rhamnolipid production, through binding to LasR protein [63].

*P. aeruginosa* infections cause mild to severe skin infections, such as green nail syndrome, hot tub folliculitis, ecthyma gangrenosum, subcutaneous nodules, and necrotizing skin and soft tissue infections [64]. Accordingly, the effect of β-carotene on skin infections was performed utilizing the DTR *P. aeruginosa-*wound-infected rat model in the current study. From wound measurements, it was observed that β-carotene ameliorated wound healing in the treated group, and the healing capacity was up to 90% at day 6. As well, the *Pseudomonas* burden severely declined in the treated group. Furthermore, histological examination of the treated group manifested regeneration of skin with few capillaries and newly formed hair follicles. In agreement with our results, the topical application of carotenoid (astaxanthin) accelerated full-thickness wound healing in mice [65]. According to Chong et al. (2022), carotenoids can promote wound healing by various mechanisms, including enhancing cell proliferation, reducing inflammation, and scavenging free radicals [66].

In summary, the antibacterial, antivirulence, and anti-QS properties of β-carotene against DTR *P. aeruginosa* are reported for the first time in this work. The β-carotene has been shown to have antipseudomonal activity against DTR *P. aeruginosa* that showed the highest pathogenicity score by inhibiting their growth as well as the production of virulence factors, such as biofilm, pyocyanin, motility, protease, gelatinase, EPS, pyomelanin pigment, rhamnolipid, siderophore, and hemolysin. Besides, *lasR*, *rhlR*, and *pqsR* QS genes were produced less frequently by treatment with 0.5 MIC of β-carotene. Additionally, β-carotene-treated rat groups revealed reduced *Pseudomonas* burdens and faster wound closure. The clinical application of β-carotene against DTR *P. aeruginosa* infection may be promising in the future.

## Data Availability

All data generated or analyzed during this study are included in this published article.
